# Investigation of health literacy status and related influencing factors in military health providers of Chinese People’s liberation Army, a cross-sectional study

**DOI:** 10.1186/s12889-022-14958-0

**Published:** 2023-01-03

**Authors:** Honghui Rong, Lu Lu, Lei Wang, Cairu Liu, Ling Zhang, Fengju Li, Dali Yi, Enyu Lei, Chuanfen Zheng, Qingbin Meng, Ji-an Chen

**Affiliations:** 1 Department of Health Education, School of Military Preventive Medicine, Army Medical University (Third Military Medical University), Chongqing, China; 2 Department of Medical Technologies and Health Care, NCO School, Army Medical University, Chongqing, Hebei China; 3 The Teaching Evaluation Centre, NCO School, Army Medical University, Chongqing, Hebei China; 4 Department of Basic Education, NCO School, Army Medical University, Chongqing, Hebei China

**Keywords:** Military health providers, Health literacy, China

## Abstract

**Objective:**

The aim of this study was to investigate health literacy and analyze its influencing factors in military health providers of the Chinese People’s Liberation Army (PLA Army).

**Methods:**

From November to December 2018, cluster sampling was used to select 1512 military health providers from the Army Medical University. Health literacy was measured by using the Chinese Citizen Health Literacy Questionnaire (HLQ) (2015 edition). Influencing factors that may affect health literacy were assessed using the chi-square test and multivariate logistic regression models.

**Results:**

The knowledge rate of health literacy was relatively low (21.6%). The knowledge rate of health-related skills (HRS, 18.7%) was the lowest of the three aspects of health literacy, and the knowledge rate of chronic diseases (CD, 19.6%) was the lowest of the six dimensions of health literacy. Participants who were older, were female, were of Han ethnicity, were the only child in their families, came from urban areas, never used tobacco, and had higher household income were likely to have higher health literacy.

**Conclusion:**

The health literacy levels of military health providers of the PLA Army are relatively low. Further research and health education are necessary to improve health literacy.

## Background

Health literacy (HL) is often defined as the degree to which an individual is able to obtain, process, and understand basic health information and services to make appropriate health decisions [1]. As a variable associated with health outcomes, health literacy is important for health behavior, health quality, and acquiring health information and health care [2–4]. Numerous studies have reported an association between low health literacy and poor health outcomes, including an inadequate understanding of diagnoses, treatment plans, and prescription drug label instructions [5, 6]; low use of preventive health services [7, 8]; lack of engagement with health care providers and knowledge about medical conditions [8, 9]; and higher risks of hospitalization and even mortality [10–12]. Because of the relationship between health literacy and health status, health literacy has been treated as a public health goal in many countries, and interventions to improve it are often prioritized [13, 14].

This is particularly true for China. In 2005, the Chinese government introduced the concept of health literacy through a manual titled “Basic Knowledge and Skills of People’s Health Literacy” [15]. In 2008, the first nationwide health literacy surveillance campaign based on a 66-item questionnaire called “Health Literacy for Chinese Citizens—Basic Knowledge and Skills” was conducted by the Chinese government [16, 17]. The result showed that only 6.48% of the 79,542 participants had adequate health literacy. The second national survey was conducted in 2012, and the survey has been conducted every year since then. The rate of health literacy rose steadily from 8.8% in 2012 to 23.15% in 2020 [18]. In 2016, the Chinese government issued its “Healthy China 2030 Blueprint”, which proposed targets to be achieved by 2030 for major health indicators, such as average life expectancy, infant mortality rate, and mortality rate of children below 5 years old. In this blueprint, the aim is to increase the rate of national health literacy to 30%, tripling the level in 2015.

Because of their intensive military activities and strict environment, military personnel bears greater health burdens than the general population, especially those engaging in military actions. After finishing vocational training, military health providers are the main force providing the primary medical service in the Chinese People’s Liberation Army. Their duty is to offer basic medical support and maintain the health of their troops [19]. This means that they are responsible for not only their own health but also the health of their comrades. Therefore, having adequate health literacy is important for this population. Some studies have reported the level of health literacy among military personnel in China [20–22]. However, to our knowledge, little is known about the health literacy of military health providers, underscoring the importance of studying this unique population and developing interventions if necessary.

The aim of this study was to evaluate the relationship between HL and health-related behaviors and background variables of military health providers. The objectives of this study are to: (1) investigate the health literacy status of military health providers in the PLA Army and (2) analyze the risk factors that affect the health literacy of this population. If risk factors can be identified, health education and promotion interventions may be used to improve their health literacy. The study findings may provide evidence to support the development of related health policies in PLA.

## Materials and methods

### Participants

In this cross-sectional survey, the target participants were military health providers from the PLA Army who were receiving vocational training in the Army Medical University, which is the main institution for educating and training military medical staff in the PLA Army. The survey was conducted from November to December in 2019. During this time, all military health providers in training in the university were invited to participate in this survey. In total, 1518 military health providers were interviewed, and 6 of them were excluded because they failed to complete the interview. Finally, 1512 interviews were included in our analyses. Before the survey, its purpose was explained to participants by well-trained investigators, and they were asked to provide verbal informed consent. Before the start of the interview, participants were asked to provide their sociodemographic information. Written informed consent was obtained from every student before providing them with the questionnaires, and the study was approved by the Ethics Review Board of the Army Medical University.

### Questionnaire

Data were obtained through face-to-face interviews using the Chinese Citizen Health Literacy Questionnaire (HLQ) (2015 edition) [16] developed by the Chinese Ministry of Health. The HLQ was widely used to investigate health literacy of Chinese population [16, 20, 23–25] and had good reliability and validity [16, 20, 24, 25]. As Chinese is the common and official language of China, the questionnaire was prepared in simplified Chinese. The questionnaire consisted of two parts: (1) demographic characteristics of the individual student (gender, age, BMI, whether they were the only child or not, ethnicity, area of residence), students’ health behaviors (smoking, alcohol use, hours per week spent playing online games), and family situation and parent-related information (annual household income, family structure) and (2) health literacy content. Based on the “Chinese Resident Health Literacy—Basic Knowledge and Skills (Trial)” and existing public health issues in China, the health literacy section (50 questions) was further categorized into three aspects and six dimensions. The three aspects were (1) knowledge and attitudes (KAA, 23 questions), (2) health-related behaviors and lifestyle (BAL, 15 questions), and (3) health-related skills (HRS, 12 questions). The six dimensions were (1) scientific views of health (SVH, 8 questions), (2) infectious diseases (ID, 6 questions), (3) chronic diseases (CD, 9 questions), (4) safety and first aid (SAFA, 10 questions), (5) medical care (MC, 11 questions), and (6) health information (HI, 6 questions). An overall health literacy score was computed as the sum of all three aspects and six dimensions.

### Evaluation method

Four types of questions were included in the scale: true-or-false questions, single-answer questions, multiple-answer questions, and situational questions. For true-or-false questions and single-answer questions, 1 point was assigned to the correct answer. For multiple-answer questions, 2 points were assigned when all of the correct answers and no incorrect answers were chosen. For situational questions, the participants were required to answer single- or multiple-answer questions after reading the given material. A score of 0 was recorded for a wrong answer. The overall health literacy score ranged from 0 to 65 points. The total scores in the three aspects KAA, BAL, and HRS were 29, 20, and 16, respectively, and the total points scored in the six dimensions SVH, ID, CD, SAFA, MC, and HI were 11, 7, 12, 14, 13, and 8, respectively. Participants who received 80% or higher of the total score (≥52) were considered to have adequate health literacy [20, 24, 25]. The knowledge rate (%) of health literacy was calculated using the following formula: total number of participants who had adequate knowledge of health literacy / total number surveyed × 100%. The knowledge rates of the three aspects and six dimensions of health literacy were calculated similarly [20, 24, 25].

### Statistical analysis

All data were input into an Epidata version 3.1 database after checking and correcting errors. SPSS 22.0 (International Business Machines Corporation, Armonk, NY, USA) was used for analyses. A descriptive analysis (frequencies, percentages, and means with standard deviations) of participant characteristics was performed. The chi-square test was used to compare the knowledge rates of health literacy among groups. Multiple logistic regression was used to assess the risk factors associated with health literacy. Statistical significance was set to *p*-value < 0.05 (two-sided).

## Results

### Sociodemographic characteristics of the participants as shown in Tables 1, 1060 of the participants (70.1%) were male and 452 (29.9%) were female

Most (85.3%) had normal BMIs. Nearly half of the participants (49.0%) lived in urban areas. The average age was 21.6 ± 1.7 years. Most participants (94.6%) were of Han nationality. Approximately 60% of the participants were not the only child in their families, and the percentage of participants who came from urban areas was 49.0%. Nearly two-thirds (65.0%) of the participants never used tobacco, and 83.5% of participants did not use alcohol. A total of 914 (60.4%) participants played online games for more than five hours per week. Nearly half (51.0%) of the participants had a family income of more than 5000 CNY per month.

**Table 1:** Sociodemographic characteristics and knowledge rate of health literacy among military health providers in PLA Army

Characteristics	N (%) ^**a**^	N (%) of Health Literacy ^**b**^	χ2	***p***
**Age**				
18–20	417 (27.6%)	45 (10.8%)	40.83	< 0.001
21–25	1079 (71.4%)	275 (25.5%)		
26 or above	16 (1.1%)	6 (37.5%)		
**Gender**				
Male	1060 (70.1%)	171 (16.1%)	61.79	< 0.001
Female	452 (29.9%)	155 (34.3%)		
**BMI** ^**c**^				
< 18.0	50 (3.3%)	9 (18.0%)	0.391	0.823
18.0–23.9	1290 (85.3%)	280 (21.7%)		
≥24.0	172 (11.4%)	37 (21.5%)		
**Ethnic**				
Han	1430 (94.6%)	319 (22.3%)	8.70	0.003
Ethnic minority	82 (5.4%)	7 (8.5%)		
**The Only Child**				
Yes	611 (40.4%)	166 (27.2%)	19.07	< 0.001
No	901 (59.6%)	160 (17.8%)		
**Area of Residence**				
Urban	741 (49.0%)	205 (27.7%)	32.02	< 0.001
Rural	771 (51.0%)	121 (15.7%)		
**Smoking**				
Yes	529 (35.0%)	53 (10.0%)	64.09	< 0.001
No	983 (65.0%)	273 (27.8%)		
**Alcohol Drinking**				
Yes	243 (16.1%)	26 (10.7%)	20.20	< 0.001
No	1269 (83.9%)	300 (23.6%)		
**Hours of Playing Online Games**				
< 5 h per week	598 (39.6%)	100 (16.7%)	13.69	< 0.001
≥5 h per week	914 (60.4%)	226 (24.7%)		
**Family Income** ^**d**^				
< 5000 CNY per month	741 (49.0%)	138 (18.6%)	7.41	0.007
≥5000 CNY per month	771 (51.0%)	188 (24.4%)		

^a^ Percentage of all participants; ^b^ number (percentage) of participants with adequate health literacy;

^c^ *BMI* body mass index, ^d^ *CNY* Chinese yuan (￥)

### The association between the knowledge rate of health literacy and sociodemographic characteristics

As indicated in Table 1, the knowledge rates of health literacy significantly differed by age, gender, ethnicity, only-child status, area of residence, smoking, alcohol consumption, hours of playing online games, and family income (*p* < 0.05), but not by BMI.

### Average scores and knowledge rates of Total health literacy and the three aspects and six dimensions of health literacy

The average scores for each health literacy scale are shown in Table 2. The knowledge rates of the three aspects KAA, HRS, and BAL were 46.7, 18.7, and 20.8%, respectively. Additionally, for the six dimensions, the knowledge rate in descending order was 44.2, 20.4, 19.6, 66.4, 35.8, and 26.5% for SVH, ID, CD, SAFA, MC, and HI, respectively.

**Table 2:** Average scores and knowledge rates of each scale in the three aspects and six dimensions

Characteristics ^a^	Mean ± SD	N of ≥80% Score ^b^	Knowledge Rate (%)
Three aspects			
KAA	20.37 ± 5.99	706	46.7
BAL	11.95 ± 4.21	314	20.8
HRS	9.11 ± 3.84	283	18.7
Six dimensions			
SVH	7.41 ± 2.62	669	44.2
ID	4.20 ± 1.60	308	20.4
CD	7.15 ± 2.75	296	19.6
SAFA	10.58 ± 3.72	1004	66.4
MC	8.10 ± 2.79	541	35.8
HI	3.98 ± 2.07	401	26.5
Health literacy	41.42 ± 12.96	326	21.6

^a^ *KAA*: Knowledge and attitudes, *BAL*: Health-related behaviour and lifestyle, *HRS*: Health-related skills, *SVH*: Scientific views of health, *ID*: Infectious diseases, *CD*: Chronic diseases, *SAFA*: Safety and first aid, *MC*: Medical care, *HI*: Health information

^b^ Number of participants with a total score ≥ 80%

### Multivariate logistic regression analysis of risk factors associated with the health literacy knowledge rate

The variables with statistical significance in the chi-square test (Table 1) were analyzed using multivariate logistic regression. As shown in Table 3, participants aged 21–25 years (OR = 2.146, 95% CI: 1.488–3.094) or more than 26 years old (OR = 4.479, 95% CI: 1.393–14.405) were more likely to have a significantly higher health literacy rate than those aged below 20 years. Compared to male participants, female participants (OR = 1.600, 95% CI: 1.155–2.215) were more likely to have a significantly higher health literacy knowledge rate. Participants who were minorities (OR = 0.381, 95% CI: 0.169–0.858) were more likely to have lower health literacy levels than participants of Han nationality. Participants who were not the only child in their families (OR = 0.613, 95% CI: 0.465–0.808) were more likely to present lower health literacy levels. Participants from rural areas (OR = 0.575, 95% CI: 0.435–0.760) were more likely to have a significantly lower health literacy knowledge rate than urban participants, and those who did not use tobacco (OR = 2.335, 95% CI: 1.619–3.367) were positively correlated with a higher health literacy level. Participants with higher average household income (OR = 1.466, 95% CI: 1.126–1.909) were more likely to present higher health literacy levels.

**Table 3:** Multivariate logistic regression analysis of influencing factors associated with health literacy of participants

Characteristics	OR	95% CI	***p***
**Age**			
18–20	1		
21–25	2.146	1.488–3.094	< 0.001
26 or above	4.479	1.393–14.405	0.012
**Gender**			
Male	1		
Female	1.600	1.155–2.215	0.005
**BMI**			
< 18.0	1		
18.0–23.9	1.331	0.602–2.941	0.480
≥24.0	1.705	0.703–4.133	0.238
**Ethnic**			
Han	1		0.020
Ethnic minority	0.381	0.169–0.858	
**The Only Child**			
Yes	1		
No	0.613	0.465–0.808	0.001
**Area of Residence**			
Urban	1		
Rural	0.575	0.435–0.760	< 0.001
**Smoking**			
Yes	1		
No	2.335	1.619–3.367	< 0.001
**Alcohol Drinking**			
Yes	1		
No	1.546	0.963–2.482	0.071
**Hours of Playing Online Games**			
< 5 h per week	1		
≥5 h per week	1.011	0.751–1.359	0.945
**Family Income**			
< 5000 CNY per month	1		
≥5000 CNY per month	1.466	1.126–1.909	0.005

## Discussion

The knowledge rate of health literacy among military health providers in this study was 21.6% (with an average score of 41.42 ± 12.96), which is a little higher than the average level of Chinese residents in 2018 (17.1%) [26] and similar to other studies that focused on the HL level among the young population [20, 24]. However, the result of the present study still reveals a large gap compared with the knowledge rate in developed areas [25]. Considering their duties, the high proportions of military health providers with limited health literacy indicate that improving the health literacy level of this population is still a challenge and should be the focus of military health education and health promotion.

Among the three aspects, the knowledge rate of health literacy related to HRS (18.7%) was comparatively lower than the other two components of comprehensive health literacy: KAA (46.7%) and BAL (20.8%). This finding may indicate that military health providers in the PLA Army lack the skills to maintain health and prevent health problems such as training injuries, which is one of the most serious health issues for military personnel [27, 28]. Further education to improve HRS among military health providers is necessary.

The knowledge rate of CD (19.0%) was the lowest of the six dimensions in the present study, consistent with other studies [24, 29, 30]. Although chronic diseases ap-pear to be much less frequent in the young population, especially in military personnel, low CD levels may become a barrier for people to improve their lifestyles, manage their diseases, and participate in complex decisions about treatment [31]. This indicates that strengthening health education and promoting health literacy are essential, particularly in terms of CD.

The results of multivariate logistic regression analysis indicate that age, gender, ethnicity, only-child status, the area of residence, smoking, and family income may affect health literacy.

In this study, age was positively associated with the level of health literacy, which is similar to the results of some studies focusing on health literacy among adolescents or young adults [30, 32]. The reason may be that as age increases, the education and health information received also increase among young adults. This may indicate that continuous health education can improve health literacy among military health providers.

Female military health providers were more likely to have adequate health literacy compared with their male counterparts. This finding is in line with previous studies [24, 32]. There are two possible explanations for this phenomenon. First, females may be more concerned about their health and be more willing to turn to others or the media for health information [33]. Second, females may be more attentive to details and care more about their personal image. These findings indicate that further education should focus on males among military health providers.

Our study results show that being an ethnic minority was associated with lower health literacy. Wang et al. reported that ethnic minorities (Hui ethnicity) had a higher rate of low health literacy than individuals of Han ethnicity [34]. Wang et al. per-formed a meta-analysis and found that Han ethnicity is a significant factor of health literacy [35]. Some foreign studies also reported that low health literacy is prevalent among racial or ethnic minorities [12, 36, 37]. A potential explanation is that ethnic minority participants were mainly from underdeveloped areas, and health literacy has been shown to be closely related to education and socioeconomic status [30]. In addition, previous studies reported that low health literacy may interact with racial/ethnic minority status to affect health status [34]. Although few of the military health providers were ethnic minorities (82 of 1512), individually targeted health education and health promotion activities are necessary for this population.

Participants who were an only child were more likely to have a higher rate of health literacy in the present study. In a longitudinal survey, Du et al. found that adolescents or young adults who were the only child in their families had higher health literacy in college [24]. An only child can receive more attention and more health-related guidance from their parents [38]. The age of participants in our study was relatively young (21.6 ± 1.7 years). Their health literacy was presumably still influenced by close parent–child relationships.

A higher knowledge rate of health literacy was observed for participants from urban areas compared to participants from rural areas, similar to previous surveys [24, 29]. A potential explanation is that participants from urban areas have easier access to high-quality health information and medical information services. Therefore, participants from rural areas should be a key focus for future health education and health promotion.

This study showed that participants who had higher family income were more likely to have a higher rate of health literacy. These findings are consistent with previous studies [20, 25, 30]. This suggests that higher health literacy levels are associated with economic factors. One reason that potentially explains this result is that with improvements in family income levels, people are more likely to attend to their own health and that of their relatives. In addition, a higher household income improves the quality of a person’s life, such as diet, which can improve health status.

Our results suggest that participants’ smoking status is a significant determinant of health literacy. The relationship between unhealthy lifestyles, such as smoking, and health literacy has been widely reported in different populations from youth to elderly [39–42]. Tobacco use is one of the most serious health risks. Therefore, implementing health education and health promotion for smoking-free is paramount in PLA.

## Limitations

There were several limitations to this study. First, due to the nature of a cross-sectional survey, it is hard to draw causal conclusions. Second, the population in this study was military health providers in the PLA Army; our results may have limited generalizability to other populations.

## Conclusions

The knowledge rate of health literacy of military health providers in the PLA Army was relatively low (21.6%). The knowledge rate of health-related skills (HRS, 18.7%) was the lowest among the three aspects, and the knowledge rate of chronic diseases (CD, 19.0%) was the lowest among the six dimensions. The knowledge rate of health literacy was positively associated with being older, being female, living in urban areas, being an only child, and having a higher family income but negatively associated with being an ethnic minority or smoking. Thus, further research and health education could be based on high-priority topics and influencing factors to improve health outcomes.

## Abbreviations

HLQ: Chinese Citizen Health Literacy Questionnaire;
PLA: Chinese People’s Liberation Army
KAA: Knowledge and attitudes;
BAL: Health-related behavior and lifestyle;
HRS: Health-related skills;
SVH: Scientific views of health;
ID: Infectious diseases;
CD: Chronic diseases;
SAFA: Safety and first aid;
MC: Medical care;
HI: Health information;
BMI: body mass index;
CNY: Chinese yuan.
